# Enzymatic Deglycation of Damaged Skin by Means of Combined Treatment of Fructosamine-3-Kinase and Fructosyl-Amino Acid Oxidase

**DOI:** 10.3390/ijms24108981

**Published:** 2023-05-19

**Authors:** Ignace De Decker, Margo Notebaert, Marijn M. Speeckaert, Karel E. Y. Claes, Phillip Blondeel, Elisabeth Van Aken, Jo Van Dorpe, Filip De Somer, Margaux Heintz, Stan Monstrey, Joris R. Delanghe

**Affiliations:** 1 Burn Center, Ghent University Hospital, C. Heymanslaan 10, 9000 Ghent, Belgium; ignace.dedecker@ugent.be (I.D.D.);; 2 Department of Plastic Surgery, Ghent University Hospital, C. Heymanslaan 10, 9000 Ghent, Belgium; 3 Department of Diagnostic Sciences, Ghent University Hospital, C. Heymanslaan 10, 9000 Ghent, Belgiumjoris.delanghe@ugent.be (J.R.D.); 4 Department of Nephrology, Ghent University Hospital, C. Heymanslaan 10, 9000 Ghent, Belgium; 5 Department of Head and Skin, Ghent University Hospital, C. Heymanslaan 10, 9000 Ghent, Belgium; 6 Department of Pathology, Ghent University Hospital, C. Heymanslaan 10, 9000 Ghent, Belgium; 7 Department of Cardiac Surgery, Ghent University Hospital, C. Heymanslaan 10, 9000 Ghent, Belgium; 8 Faculty of Medicine and Health Sciences, Ghent University, Sint-Pietersnieuwstraat 33, 9000 Ghent, Belgium

**Keywords:** fructosamine-3-kinase, fructosyl-amino acid oxidase, advanced glycation end products

## Abstract

The consensus in aging is that inflammation, cellular senescence, free radicals, and epigenetics are contributing factors. Skin glycation through advanced glycation end products (AGEs) has a crucial role in aging. Additionally, it has been suggested that their presence in scars leads to elasticity loss. This manuscript reports fructosamine-3-kinase (FN3K) and fructosyl-amino acid oxidase (FAOD) in counteracting skin glycation by AGEs. Skin specimens were obtained (n = 19) and incubated with glycolaldehyde (GA) for AGE induction. FN3K and FAOD were used as monotherapy or combination therapy. Negative and positive controls were treated with phosphate-buffered saline and aminoguanidine, respectively. Autofluorescence (AF) was used to measure deglycation. An excised hypertrophic scar tissue (HTS) (n = 1) was treated. Changes in chemical bonds and elasticity were evaluated using mid-infrared spectroscopy (MIR) and skin elongation, respectively. Specimens treated with FN3K and FAOD in monotherapy achieved an average decrease of 31% and 33% in AF values, respectively. When treatments were combined, a decrease of 43% was achieved. The positive control decreased by 28%, whilst the negative control showed no difference. Elongation testing of HTS showed a significant elasticity improvement after FN3K treatment. ATR-IR spectra demonstrated differences in chemical bounds pre- versus post-treatment. FN3K and FAOD can achieve deglycation and the effects are most optimal when combined in one treatment.

## 1. Introduction

The global market value of cosmeceuticals helping people fend off the consequences of natural aging is continuously increasing and is expected to reach a market value of 805.61 billion dollars in the U.S. by 2023 [1,2]. Aging is an extremely complex chronic process spanning many years and can be subdivided into intrinsic (chronological) and extrinsic (e.g., photoaging, pollution, and smoking) aging [2,3,4]. Many different hypotheses have been generated over the years to reveal the causality of skin aging, and the general consensus is that inflammatory processes, cellular senescence, free radicals, and epigenetic changes play crucial roles [5,6,7,8]. It has been demonstrated that advanced glycation end products (AGEs) play an indispensable role in skin aging and are linked with the skin’s physiology and functioning [9,10]. Moreover, it has been reported that these irreversible products play a role in the dermal wound healing process by inducing apoptosis in various cell types, leading to deteriorated healing [11,12]. Additionally, it has been suggested that owing to the interaction with various cell types in the skin and its extracellular matrix, AGEs are responsible for shortened, thinned, and disorganized collagen fibers, which results in the loss of the elasticity of scar tissue [9,13,14,15,16].

The formation of these AGEs is the result of a non-enzymatic reaction between either a free amino acid or a protein-bound amine and the carbonyl groups of reducing sugars, which is called the Maillard reaction [1,17,18]. These early stage products are called Schiff bases and, if rearranged in a stable manner, Amadori products [18,19]. Schiff bases can also produce highly reactive intermediates such as glycolaldehyde or glyoxal [18]. The Amadori products undergo complex rearrangements which consist of sequential condensations, oxidative modifications, cleavage, and covalent binding reactions, that can happen over weeks or months until eventually irreversible end products are created, known as AGEs. AGEs are a heterogeneous group of substances consisting of stable adducts and crosslinks of proteins [1,9,18]. AGEs can be divided into fluorescent and non-fluorescent proteins; examples include pentosidine and carboxymethyllysine, respectively [18,20]. The accumulation of AGEs is the most apparent in proteins with a long-lasting half-life; therefore, it is understandable that structures such as albumin, hemoglobin, low-density lipoprotein cholesterol, collagen, and elastin are well-known targets of these eventual irreversible modifications caused by glycation [1,9,17,21]. Proteins that are excreted or rapidly broken down are not prone to this conversion [9]. AGEs accumulate in the body over a person’s lifetime in both intracellular and extracellular environments [17]. Intracellularly, AGEs can bind to specific AGE receptors (RAGE), thereby upregulating proinflammatory cytokines and matrix metalloproteinases (MMPs) and increasing the production of reactive oxygen species (ROS) [3]. Receptors for AGEs have been detected in vivo on the surfaces of keratinocytes, endothelial cells, melanocytes, and fibroblasts [9]. At the extracellular level, protein crosslinking due to AGEs can inhibit cell growth, impair cell adhesion, and cause overall tissue dysfunction [17]. Several age-related diseases such as Alzheimer’s disease, diabetes mellitus, atherosclerosis, and dialysis-related amyloidosis are thought to be related to AGE accumulation [19]. Owing to the accumulation of AGEs, human aging leads to skin stiffening. This is due to damage to collagen fibers caused by Maillard reactions [22]. Moreover, it has been elaborately described that AGEs play an important pathological role in the dermal wound healing process and the current evidence on the role of AGEs in scar formation indicates that a higher accumulation of these products tends to produce more rigid, contractile scars with a persistent erythematous appearance [12,23].

The accumulation of AGEs occurs from as young as the age of 20 years and increases steadily over time [9]. AGEs such as pentosidine and carboxymethyllysine have been demonstrated to accumulate in both the epidermal and dermal layers of the skin [8,17]. This aggregation leads to dermal aging and is slow to appear, but is known to cause a stiffening of collagen, elastin, and fibronectin [4,17,24]. In other words, it results in permanent damage, and clinical signs become noticeable when the deceleration of protein expression can no longer outweigh the rate of protein degradation [4]. Structural damage and degradation of these connective and supportive proteins are directly associated with the impairment of the skin’s physiological tissue structure, leading to the formation of skin wrinkles and loss of elasticity [21,25]. Moreover, AGE-RAGE activation promotes an increase in melanin production through the induction of melanogenesis [9,17]. Due to the same receptor interaction, keratinocytes gradually lose their proliferation properties, and fibroblasts lose contractility and their turn-over capabilities [9]. All of these factors are detrimental to the youthful appearance of the skin. Therefore, deglycation compounds and glycation inhibitors are of immense research interest for the treatment of aging [21,26,27].

Many researchers have attempted to identify bioactive compounds that either inhibit AGE formation or reverse this irreversible process [1,28,29,30]. In the current manuscript, we report the deglycation properties of two enzymatic compounds: fructosamine-3-kinase (FN3K) and fructosyl-amino acid oxidase (FAOD), and their potential therapeutic use in counteracting skin glycation.

## 2. Results

### 2.1. Autofluorescence of GA-Modified and Enzymatically Treated and Untreated Skin

**Figure 1:**
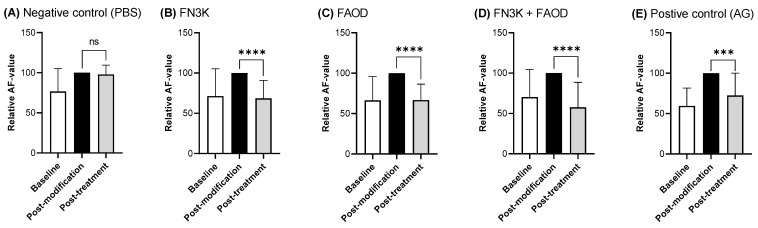
Relative autofluorescence (AF) values of glycolaldehyde-induced AGE-modified skin samples (n = 19) treated with deglycation enzymes, FN3K (**B**), FAOD (**C**), and a combination of FN3K and FAOD (**D**). PBS was used as a negative control (**A**) and AG was used as a positive control (**E**). Statistical significance was defined as *p* < 0.05. ‘***’ = *p* < 0.001. ‘****’ = *p* < 0.0001. ‘ns’ = not significant.

Figure 1 shows the relative AF values of GA-induced AGE-modified skin after PBS (control), FN3K, FAOD, FN3K+FAOD, and AG treatment. A significant decrease in AF values was observed for each of the three enzymatic treatments. In FN3K skin, an average decrease of 31% in AF was observed between baseline and after 6 h of treatment. In addition, for FAOD-treated skin, a similar average decrease of 33% in AF was detected between the baseline and 6 h of treatment. A more pronounced average decrease of 43% in AF was observed in the skin treated with both FN3K and FAOD. The negative control showed no significant differences in AF between the baseline and after 6 h of PBS treatment. The positive control showed a 28% decrease in AF between baseline and 6 h of treatment.

### 2.2. Elasticity Measurements of Hypertrophic Scar Tissue

**Figure 2:**
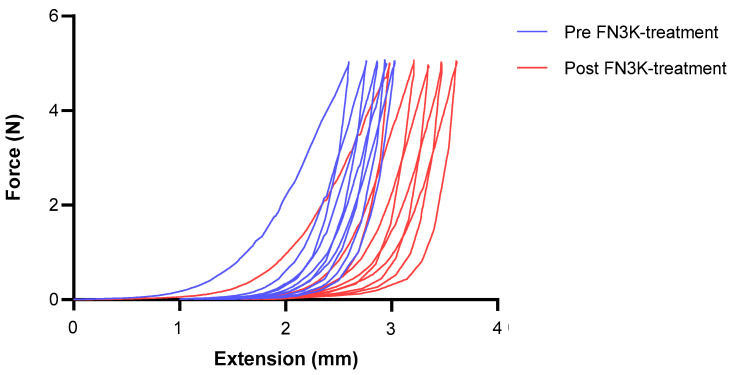
Elongation rate of hypertrophic scar tissue with a thickness of 12 mm before and after FN3K treatment. ■: Pre-FN3K treatment. ■: Post-FN3K treatment.

An elasticity measurement of the hypertrophic scar with a loading force of up to 5 N over five cycles from extension to the peak (Figure 2) showed a significant difference in elasticity before (elongation, 3.205 ± 0.0733 mm; *p* ≤ 0.001) and after (elongation, 3.622 ± 0.114 mm; *p* ≤ 0.001) FN3K treatment. The degree of elongation increased by approximately 13% after treatment.

### 2.3. MIR-Spectroscopy Analysis of Treated Hypertrophic Scar Tissue

**Figure 3:**
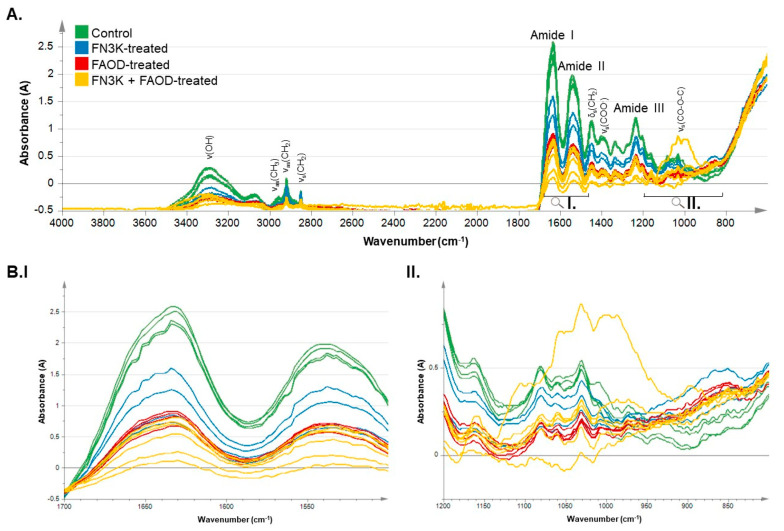
(**A**) Normalized ATR-IR spectra of hypertrophic scar tissue after PBS (control), FN3K, FAOD and FN3K + FAOD treatment showing typical skin MIR peaks and indicated regions of interest over a range of 4000−400 cm^−1^. (**B**) Magnification of the regions of interest including (**I**) the amide peaks I and II (proteins) and (**II**) the carbohydrate region. ■: control, ■: FN3K-treated, ■: FAOD-treated, ■: FN3K + FAOD-treated. s: Symmetrical vibrations, as: asymmetrical vibrations, δ: bending vibrations and v: stretching vibrations.

Figure 3A shows the MIR spectra of hypertrophic scar tissue after PBS (control), FN3K, FAOD, and FN3K + FAOD treatments. Figure 3B(I) shows an enlargement of the characteristic region of the amide peaks I and II (1500–1690 cm^−1^), showing a decrease in the peaks between the control and treated skin. First, a decrease in the peak of the FN3K-treated scar, followed by another decrease in the peak of the FAOD-treated scar was observed. Finally, FN3K+FAOD-treated hypertrophic scars showed the greatest reduction, despite very minor differences. In the carbohydrate region (900–1200 cm^−1^), as shown in Figure 3B(II), there was, although less pronounced, an analogous decrease in the amide region. Again, the spectrum of the FN3K+FAOD-treated scar showed the lowest peaks. The spectra of both the FN3K- and FAOD-treated scars were between those of the control and FN3K + FAOD-treated scars.

**Figure 4:**
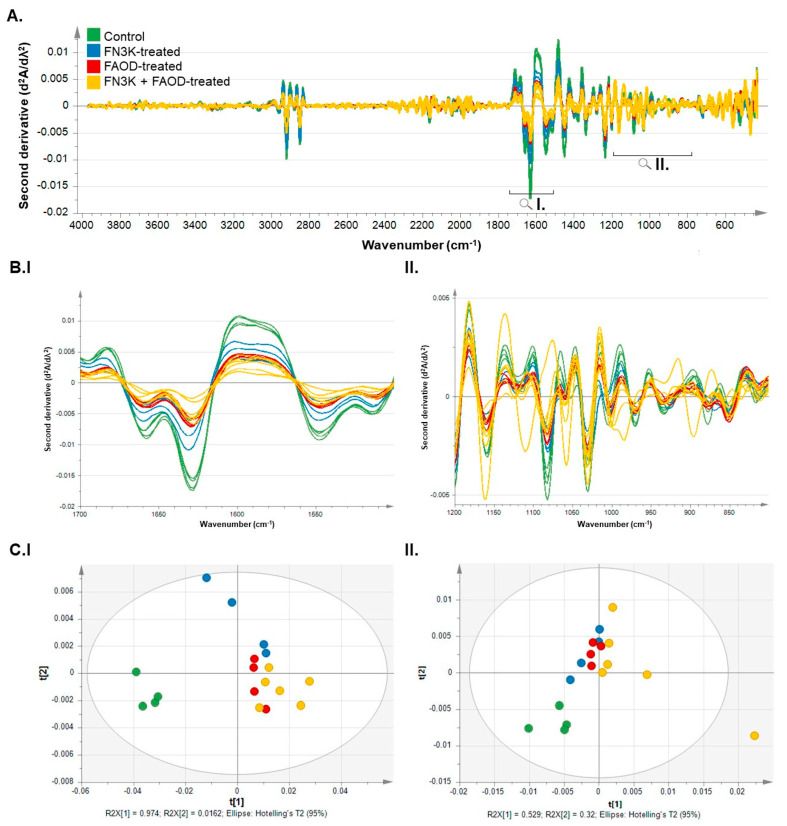
(**A**) Second derivative of the preprocessed MIR spectra (SNV and SG smoothing: 15 points) of hypertrophic scar tissue after PBS (control), FN3K, FAOD, and FN3K + FAOD treatment over a range of 4000–400 cm^−1^. (**B**) Magnification of regions of interest including (**I**) the amide peaks I and II (proteins) and (**II**) the carbohydrate region. (**C**) Score plot of a PCA analysis of amide peaks I and II (**I**) and the carbohydrate region (**II**). ■: Control, ■: FN3K-treated, ■: FAOD-treated, ■: FN3K + FAOD-treated.

The second derivative of the pre-processed spectra, shown in Figure 4, also demonstrated differences between the control and treated scar tissues. In the magnified regions, such as those of amide peaks I and II shown in Figure 4B(I), we again saw that peaks in the spectra of both the FN3K- and FAOD-treated scar tissue were lower than the control but higher than the FN3K + FAOD-treated scar. In the carbohydrate region shown in Figure 4B(II), these differences were less noticeable. Upon considering the PCA score plots of both the amide (Figure 4C(I)) and carbohydrate regions (Figure 4C(II)), we observed a clear distinction in the clustering between the spectra of the control and treated scar tissues. Fewer marked differences were found between the FN3K, FAOD, and FN3K+FAOD-treated spectra.

## 3. Discussion

The pathophysiology of aging and its potential prophylactic and curative treatments are common fields of interest in numerous medical specialties, including plastic surgery and dermatology, with considerable economic significance and ever-increasing interest in the pharmaceutical and cosmetic industries [31,32,33,34]. FN3K and FAOD have been reported to be useful as deglycation agents, as discussed above. In this study, the deglycation capability of FN3K and FAOD, with or without combining both enzymatic compounds, was assessed using ex vivo models for both healthy skin and hypertrophic scar tissue. 

The experimental setup of the chemically modified healthy human skin samples described in Section 2.1, shows the effectiveness of both enzymes of interest, namely FN3K and FAOD, in skin deglycation. This was demonstrated by the significant decrease in AF, following treatment for 6 h, after the initial increase in AF due to GA modification. The AF values of the samples treated with FN3K and FAOD returned to the baseline values. In contrast, the combination treatment with both enzymes resulted not only in the restoration of the initial AF values, but also in the further deglycation of the skin, reaching the highest average AF decrease of 43%. This illustrates the beneficial effects of combined enzymatic treatment. AG, which is frequently used as a positive control in antiaging experiments, was less effective in reducing AF post-modification, and although AF values decreased significantly, they did not return to baseline. The successful deglycation of the skin, as seen here, is in line with the previously reported results by De Bruyne et al., who studied the deglycation of lens material using FN3K for the treatment of cataract and age-related macular degeneration [18,35]. The present results demonstrate for the first time that both FN3K and FAOD are successful in the acute disruption of skin AGEs. Prevention of AGE production through the removal of ketoamines in the FN3K-catalyzed pathway was already known and is of great importance for future potential therapeutic applications since this implies the enzyme’s ability of ‘off the shelf’ prevention, and when needed, even reverse the damage [35].

Furthermore, by using MIR, it has been demonstrated that treating HTS results in spectroscopic changes. The localization of the differences in wavelength intensities showed changes in the carbohydrate region and amide I and II peaks. The Hotelling plots (Figure 4), which are the condensed results of the spectral differences derived from the MIR spectra, showed clear differences between treated and untreated HTS specimens following deglycation treatment [18,35].

A recent publication by Moraes et al. (2023) compared the skin parameters between a diabetic and healthy population using bio-functional measurements and reflectance confocal microscopy [22]. The authors showed that the skin quality was altered. This is related to collagen-based AGE crosslinking, where these important fibers undergo structural and functional changes and eventually become bridled and dysfunctional [13,36]. This was linked to both a loss of elasticity and a greater presence of wrinkles [22]. In this study, elongation testing of the excised HTS specimen demonstrated that with skin deglycation and the reversal of AGE-induced crosslinking by use of FN3K, decreased scar rigidity can be obtained. Therefore, treatment with deglycation enzymes may improve scar quality in terms of elasticity and suppleness. Furthermore, a recent study found a link between pigmentation and erythema on the one hand and AGEs on the other [10]. This is in line with previous research describing the AGE-induced promotion of melanogenesis through melanocyte receptor stimulation eventually leading to pigmentation disorders [10,37]. The effects of FN3K and FAOD on skin color, including erythema and pigmentation factors, in both anti-aging and scar improvement settings, should be the subject of future research.

The results produced in this manuscript are promising for future anti-aging and scarring treatments. However, additional data must be gathered to reproduce and (re)confirm these research models and verify the regions and magnitude of the molecular changes resulting from treating these tissues.

## 4. Material and Methods

### 4.1. Ethics Committee

The Ethical Review Committee of Ghent University Hospital approved the tissue collection and study protocol (Belgian registration number: BC-05726).

### 4.2. Fructosamine-3-Kinase

FN3K phosphorylates both free and protein-bound fructosamines on the third carbon of its sugar fraction, resulting in instability and subsequent detachment from the proteins with concomitant regeneration of the unglycated amine [18]. The currently available literature suggests that FN3K is part of the natural repair mechanism of cells and that it plays an important role in controlling non-enzymatic protein glycation [18,38,39,40]. This control mechanism is even more distinct in proteins with a longer half-life, such as collagen, elastin, crystallin, and hemoglobin [18,38,39,40]. The enzymatic compound FN3K, therefore, prevents AGE formation and has been successfully used in the deglycation of several tissues, including nails, heart valves, and lenses [18,41,42,43]. FN3K was recombinantly produced from *P. pastoris* in a previous study by De Bruyne et al. (2020) [35].

### 4.3. Fructosyl-Amino Acid Oxidase

FAOD is a defructolyzing enzyme which catalyzes the oxidation of C–N bonds that links the C1 of the fructosyl fraction and nitrogen of the amino group of the fructosyl-amino acids [44]. Recombinant FAOD (*E. coli*) sourced from *cryptococcus* with recombinant expression (DIA409, Creative Enzymes, Shirley, New York, NY, USA) was used for the experiments reported in this manuscript.

### 4.4. Aminoguanidine

Aminoguanidine (AG) is a hydrazine derivative and has been used as a representative compound for glycation inhibition and has served as a positive control in numerous experimental setups [20,21,45,46,47]. AG hydrochloride (Sigma-Aldrich, St. Louis, MO, USA) was used as a positive deglycation agent. Given the known side effects shown in previous studies, AG is not used for clinical purposes [45,47].

### 4.5. Age Modification and Enzymatic Deglycation

Healthy human breast skin was obtained from 19 female patients (mean age 26.05; standard deviation 9.02) undergoing subcutaneous mastectomies as a part of their gender-affirming surgery. Subcutaneous adipose tissue and residual fascia were carefully resected. Subsequently, this healthy skin was incubated for 3 h in a 25 mM glyceraldehyde dimer—PBS solution (crystalline form, Sigma-Aldrich) at 37 °C, as glycolaldehyde (GA) is known as a reactive intermediate during AGE formation, and thus a good component for protein modification. After incubation, the active components were washed with PBS by placing the skin in an ultrasonic bath (Branson 3510MT, Danbury, CT, USA) for 60 min. The skin was then placed overnight in fresh PBS at 4 °C to terminate the chemical reaction. Finally, the skin was placed a second and final time in an ultrasonic bath for another 60 min to remove any residual GA.

Three different enzymatic conditions were tested for the in vitro glycation reaction. For the first condition, a 1:1 solution of 250 µg/mL FN3K and cofactors (5 mM ATP-2 mM MgCl2 solution) was used as described by De Bruyne et al. (2020). Next, a 3:1 9 mg/mL FAOD and cofactor FAD solution in PBS (Sigma-Aldrich, Tokyo, Japan) were added, and finally, for the third condition, a 1:1 mixture of the FN3K and FAOD solutions was added. For glycation treatments and AF measurements, the skin fragments were transferred to a black 96-well plate (FluoroNunc PolySorp, Thermo Fisher Scientific, MA, USA), and 20 µL of the final enzymatic solution was added and incubated for 6 h at 37 °C. As a negative and positive control experiment, a skin fragment was processed in exactly the same manner but received a PBS 5 mM ATP 2 mM MgCl2 or AG treatment instead of a deglycation enzyme solution. The first AF measurements were made moments before administration of the enzymatic treatment and then measured after 3 h and 6 h of incubation.

### 4.6. Autofluorescence Measurements on AGEs

For the detection and measurement of AGE-related autofluorescence (excitation 370 nm, emission 390–700 nm) in the human skin samples, a Flame miniature spectrometer (FLAME-S-VIS-NIR-ES, 350–1000 nm, Ocean Optics, Dunedin, FL, USA) coupled to an LSS-LED light source (365 nm, Ocean Optics) and a reflection probe (QR400-7-IS-BX, Ocean Optics) was used. For the AF measurements, first, the background signal was corrected, after which the skin samples were transferred to a black 96-well plate (SPL Lifesciences, Pochon, Republic of Korea), and fluorescence measurements were taken at a 90° angle. Each measurement was repeated three to five times. The intensity of the light source was manually set to an excitation peak with an intensity of approximately 60,000. The AF measurements were processed using the OceanView program (Ocean Optics, Largo, FL, USA), which was set to an average of 128 scans and an integration time of 10 ms. The AF values were calculated by dividing the mean light intensity emitted per nm for a range of 407–677 nm by the mean light intensity per nm for a range of 342–407 nm [35,48]. 

### 4.7. Elasticity Testing of FN3K-Treated Hypertrophic Scar Tissue

Loss of elasticity is a known effect of AGEs on the skin. As hypertrophic scar tissue is known to be one of the most rigid skin tissues, we investigated whether treatment with a deglycation enzyme, FN3K, could provide a gain in elasticity. A hypertrophic scar resulting from a thermal burn was resected and treated ex vivo with topical FN3K solution. Before and after the FN3K treatment, elasticity measurements were performed using an LFPlus Universal material tester (Lloyd Materials testing, Bognor Regis, UK) equipped with a descending ball probe with a diameter of 4.45 mm and a compression speed of 10 mm/min. As a zero indentation point, the lower limit of the force was set to 0.001 N for a piece of hypertrophic scar with a thickness of 12 mm. In addition, a 10 N load cell was used, and a maximum force of 5 N was chosen to avoid tissue damage. Finally, five depressions were performed to exclude the hysteresis effect. Thus, the stiffness parameters (N/mm) of both the treated and untreated scar areas were determined.

### 4.8. Mid-Infrared Spectroscopy and Multivariate Data Analysis

Mid-infrared spectra of the same hypertrophic scar tissue as described above, both treated and untreated, were obtained by attenuation of the total reflectance-Fourier transform infrared (ATR-FTIR) spectroscopic analysis of uncovered, deparaffinated sections with a thickness of 10 µm. Each section contained several cross-sections of the skin sample which were each measured once, using a Perkin Elmer Spectrum Two ATR -FTIR spectrometer equipped with the Perkin Elmer Universal ATR Accessory (a zinc selenide (ZnSe) crystal) and Spectrum 10 software (Perkin Elmer, Waltham, MA, USA). The MIR-spectra were preprocessed and analyzed by multivariate analysis using the SIMCA^®^ software version 15.0.2 (MKS Data Analytics Solutions, Umeå, Sweden). To standardize the spectral data, they were preprocessed using different spectral filters. The spectra were normalized using the standard normal variation (SNV), converted to their second derivative, and subjected to the Savitzky–Golay (SG) algorithm (15 smoothing points). SNV removes variations in the baseline caused by measurement variations between different samples. Spectral differentiation can be used to highlight and distinguish small structural differences between the spectra. Conversion to the second derivative mainly examines the changes in the rate of absorbance change. Finally, SG smoothing can be used to reduce the amount of noise while preserving spectral details [49,50]. The MIR spectra were recorded over a range of 4000–400 cm^−1^ at a spectral resolution of 4 cm^−1^ (five co-added scans). Smaller regions with valuable spectral peaks were further analyzed using principal component analysis (PCA).

### 4.9. Statistical Analysis

Statistical analyses were performed using GraphPad Prism version 9 (GraphPad Software Inc., San Diego, CA, USA). All data were considered to be normally distributed using the Shapiro–Wilk test. Normally distributed data are presented as mean ± standard deviation (SD). Pairwise comparisons between two normally distributed groups were performed using paired *t*-tests. Statistical significance was set at *p* < 0.05.

## 5. Conclusions

FN3K and FAOD are both capable of achieving skin deglycation and when combined in one application, the effect is the most potent, owing to a synergistic effect. Although the results are preliminary, these enzymes are promising potential treatment options for skin deglycation. This could open up possibilities for anti-aging treatments and may be effective in improving scar elasticity. Future studies should verify the success of enzymatic skin glycation in vivo.

## Data Availability

Access to the data is restricted to the research team and authorized personnel for the purpose of ensuring data integrity. However, upon reasonable request, and subject to applicable legal and ethical considerations, anonymized and aggregated data may be made available for verification and replication by qualified researchers.
